# Care transitions for frail, older people from acute hospital wards within an integrated healthcare system in England: a qualitative case study

**DOI:** 10.5334/ijic.1175

**Published:** 2014-03-27

**Authors:** Lesley Baillie, Andrew Gallini, Rachael Corser, Gina Elworthy, Ann Scotcher, Annabelle Barrand

**Affiliations:** Faculty of Health and Social Care, Florence Nightingale Foundation Chair of Clinical Nursing Practice, London South Bank University and University College London Hospitals, London, UK; Nursing for the Hospital of St John & St Elizabeth, London, UK; Nursing and Clinical Services, CareUK, Reading, UK; University of Bedfordshire, Oxford House Campus, Aylesbury, UK; University of Bedfordshire, Oxford House Campus, Aylesbury, UK; University of Bedfordshire, Butterfield Campus, Luton, UK

**Keywords:** vertical integration, integrated care, frail older people, care transitions, acute hospital wards, community healthcare

## Abstract

**Introduction:**

Frail older people experience frequent care transitions and an integrated healthcare system could reduce barriers to transitions between different settings. The study aimed to investigate care transitions of frail older people from acute hospital wards to community healthcare or community hospital wards, within a system that had vertically integrated acute hospital and community healthcare services.

**Theory and methods:**

The research design was a multimethod, qualitative case study of one healthcare system in England; four acute hospital wards and two community hospital wards were studied in depth. The data were collected through: interviews with key staff (*n* = 17); focus groups (*n* = 9) with ward staff (*n* = 36); interviews with frail older people (*n* = 4). The data were analysed using the framework approach.

**Findings:**

Three themes are presented: Care transitions within a vertically integrated healthcare system, Interprofessional communication and relationships; Patient and family involvement in care transitions.

**Discussion and conclusions:**

A vertically integrated healthcare system supported care transitions from acute hospital wards through removal of organisational boundaries. However, boundaries between staff in different settings remained a barrier to transitions, as did capacity issues in community healthcare and social care. Staff in acute and community settings need opportunities to gain better understanding of each other's roles and build relationships and trust.

## Introduction and background

Integrated health and social care services, providing a seamless service to deliver integrated care for patients and carers, are a well-established aspiration in English health policy [1–4]. Integration is the combination of methods, processes and models that aim to achieve integrated care, which is an organising principle for care delivery that aims to improve patient care through better coordination [5]. Different types of service integration have been defined in England; ‘horizontal’ integration, where organisations or services delivering similar care integrate, and ‘vertical’ integration, which brings together organisations that deliver different services, such as acute hospitals and community providers [6]. Integrated care could particularly benefit frail older people who may be in contact with many health and social care professionals for different health conditions [7] and undergo frequent care transitions between different services [8–12]. There are varied discourses about the concept and meaning of frailty [13–14]. This study used Bauer's definition [15], which concurred with understandings of frailty within the study setting:

older people who are no longer able, fully and adequately, to care for themselves because of either normal age-related changes in the body, which impair functional ability, or one or more medical conditions which, similarly, can impede activities of daily living. (p. 1173).

There are many health problems associated with frailty [16]. Therefore, as in many other countries, frail older people are a major group of health service users in England: 65% of patients admitted to hospital are over 65 years, of whom an increasing number are frail, and 25% of inpatients have dementia [17]. In frailty, the age-related decline in many physiological systems together, leaves the person vulnerable to sudden health status changes, triggered by minor stressors [18], which may lead to care transitions, often starting with an emergency hospital admission.

While many studies have investigated care transitions, particularly for older people, there are fewer studies based within England, possibly because of assumptions that transition problems are lessened within a universal health system [9]. However, care transitions remain a problematic area of policy and practice within England [11, 19] as well as internationally [20]. Few studies have been based in vertically integrated healthcare systems, yet these could potentially reduce barriers to care transitions between acute and community healthcare as traditional service boundaries are removed. The study, reported here, investigated transitions of frail older people from acute hospital wards to other non-acute healthcare services within a healthcare system in England that had vertically integrated hospital and community healthcare services.

Care transitions are complex and multidimensional in nature with varying types and patterns, and facilitating and inhibiting conditions [21]. Transition types include transfers from home-to-hospital, hospital-to-home, hospital-to-skilled care facility, and skilled care facility-to-home and/or homecare [20]. People are more vulnerable to risks that may affect their health during transitions [21] and so the quality of care related to transitions is important [22]. Transitional care provides coordination and continuity of healthcare when patients transfer between different locations or between different levels of care within the same location [22]. High-quality transitional care is especially important for older adults with multiple chronic conditions and complex care management, as well as for their family carers [23].

Research about care transitions has often focused on transition of older patients from hospital to home [23]; this critical transition point can affect health outcomes but patients remain vulnerable to poor quality, fragmented care [11, 24, 25]. While transition can be a physical, psychological and social process for older people, the latter two dimensions may be neglected by service providers [11], which may contribute to poor transitional care experiences. Discharge of frail older people from hospital is complex and multifactorial [26] and in England, concerns have exacerbated due to the increasing frailty of older people, along with more rapid throughput in acute hospitals [10, 27].

From a service perspective, delayed transfers of care continue to challenge the health and social care sector in England [19, 28] as well as other countries, such as Sweden, Norway, New Zealand and the United States (US) [29]. In England, a delayed transfer of care is defined as being when a patient is still occupying an acute hospital bed but is clinically ready and safe for transfer [30]. Some older patients who experience delayed transitions develop complications, such as infections [28], further highlighting the vulnerability of this patient group. Many policies and initiatives in England have aimed to address delayed discharge from hospital but causes are diverse and whole systems approaches are necessary [27, 31].

Guerin et al. reviewed studies of how community services can work with hospitals across the hospital–community interface and identified four models [32]. The ‘Virtual interface model’, most suitable for straightforward discharges, maintained the traditional approach of staff staying in their respective hospital or community environments and communicating through telephone or written communication, and hospital staff planning discharges and referring to community staff [32]. However, practitioners in different settings often operate independently, with little knowledge of other settings [22, 33] and deficits in communication and information transfer at hospital discharge are common [24, 34, 35] with no established process for information exchange between settings being an identified barrier [33]. The ‘In-reach Interface Model’, where community services are located in the acute care sector and are involved in discharge earlier, could be more suitable for older adults with complex discharge needs [32]. With the ‘Out-reach interface model’ hospital staff implement aspects of the discharge plans in the community, a model that could be suitable for older adults with specialised needs [32]. Finally, the ‘Independent Interface Model’ involved an independent person, not employed by the hospital or community service, working across the interface to facilitate discharge, a model that may be appropriate for older adults with complex discharge needs [32]. The models are tentative as they were identified from few studies but they indicate that there are alternative models for frail older people's care transitions between hospital and community, than the traditional ‘Virtual interface’ model.

Graham et al. studied the transitional care needs of vulnerable older people in the US and identified five levels to be considered: (1) the individual; (2) the interpersonal; (3) the organisational; (4) the community environment; and (5) policy [36]. In practice, there may be facilitators or barriers at any of these levels, which also interrelate. For example, organisational factors and related communication issues have posed barriers to how health professionals work interpersonally with individual patients during care transitions [9, 24, 35, 37]. Organisational issues include system inflexibility [9], healthcare system fragmentation and lack of standardised processes [24] and lack of timely, accurate and complete communication between services [24, 35]. However, healthcare professionals have used various strategies to improve continuity, for example, building relationships with healthcare professionals in other settings and using informal communication systems [33].

The level of patient involvement in care transition processes is important for successful transitional care [21, 22]. However, international studies reveal poor communication with patients that adversely affected transition experiences [9, 11, 20, 38, 39]. In addition, family inclusion and effective communication within the interprofessional team and with the family are key factors in successful discharge of frail older people [26], but discharge processes may not include informal carers [23, 25] and communication with carers may be lacking [27, 39]. The way older people are treated by staff has been found to have a major impact on their overall experience [11], but few standards focus on the experiences of older adults during transfers [23]. Older adults moving between home and different institutions have reported feeling unsupported, unheard and treated with insufficient dignity [9], feeling like ‘passive bystanders’ in their hospital care [39] and feeling disorientated, worried, afraid and uncertain [11]. Building relationships between practitioners and frail older people, and their relatives [10], and empowering patients during care transitions [39], could support more positive transition experiences.

In summary, frail older people are a major group within hospitals and they experience frequent care transitions. Internationally, research studies have revealed that care transitions for frail older people can be problematic. A healthcare system that has removed organisational boundaries between hospital and community healthcare through vertical integration could support smoother care transitions within the system for all patients, including the frail older population. However, there is a lack of previous research about care transitions based in the context of a vertically integrated system.

## Study aim

The study's aim was to investigate the care transitions of frail older people from acute hospital wards to community healthcare or community hospital rehabilitation wards, within the context of a healthcare system that had vertically integrated acute hospital and community healthcare services.

## Methodology

The research design was a qualitative case study. A case study design is suitable for studying a contemporary issue within context and where the boundaries of a phenomenon (in this case, care transitions), and the context (the integrated healthcare system), are not clear [40]. Following Yin's case study framework [40], the ‘case’ was one vertically integrated public healthcare system in a rural area in Southern England. Two years prior to the study's commencement there was a vertical integration of the area's two acute hospitals, and their local community healthcare services, which included community hospitals that delivered rehabilitation services and community-based healthcare teams that delivered care at home. The case study was an instrumental case study [41] which entails an in-depth investigation in order to enable a better understanding of a theoretical problem; in this case, how a vertically integrated health system facilitates care transitions for frail older people from acute hospital wards. In an instrumental case study, the particular case is selected to gain understanding of the topic studied (care transitions of frail older people) rather than the case itself.

Using Yin's framework [40], the study included embedded units of analysis (four acute hospital wards and two community hospital wards) that were studied in depth to gain understanding of care transitions. The wards were selected with expert guidance from a senior nurse, the aim being to include wards that regularly admitted frail older people and that were based in each of the two acute hospitals. The two community hospital wards were selected as they regularly admitted frail older people who were transferred from the four acute wards.

## Methods

The data were collected from July to October in 2012, through audio-recorded semi-structured interviews (key staff and patients), and focus groups (ward staff). Researchers used topic guides developed from the literature review.

A purposive sampling approach was used to identify key staff, who were invited for individual interviews via email. A senior nurse identified potential participants according to the following criteria:

- staff with direct, or strategic, involvement with planning and/or managing transitions of frail older people from the acute wards;
- a range of disciplines (medical, nursing and allied health professionals);
- staff from different locations: both acute hospitals, community hospitals and community healthcare teams.

After the first 10 interviews, the sample was reviewed in relation to the above criteria and a further group of staff were identified and invited. The final sample of 17 staff included 9 who were community-based including: a lead general practitioner, an adult community healthcare lead, district nurses, a community physiotherapist, a community occupational therapist and a senior nurse with responsibility for the community hospitals. The other eight participants were acute hospital-based and included: senior nurses, senior operational leads and staff with hospital-wide roles in care transitions. Interviews lasted approximately 30 minutes each. Researchers used open questions, based on the topic guide, to explore participants’ views about, and experiences of: the system's strategic commitment to transitions of frail older people, transition processes including involvement of patients/families, and barriers and facilitators to safe and timely transitions. Probing questions were used to elicit more in-depth responses.

The researchers aimed to conduct a focus group on each ward to explore staff experiences. Focus groups are a form of group interview in which the group interaction is explicitly used as part of the method [42]. Table 1 summarises the focus groups and participants, who were based on, or linked to, the selected wards (A–F).The original intention was to hold one focus group on each ward with a multidisciplinary staff group. However, staffing issues and time pressures on staff led to organisational difficulties. The researchers were flexible about rearranging dates and times and they ran the groups on Wards A, E and F on two occasions with small numbers so that more staff could participate. In total, 36 staff participated in the 9 focus groups, each of which lasted 30–60 minutes. The topics explored through open questions and probes were: perceptions of the system's strategic commitment to transitions of frail older people, care transition planning processes, facilitators and barriers to transitions, and patient and family involvement in planning transitions.

The project team aimed to interview patients (and their relatives if willing) who met the following criteria: frail, 70 years or above, had experienced a care transition from a selected acute ward to either a community hospital ward or home with community healthcare team support, have the mental capacity to give informed consent, and be able to communicate verbally and in English. However, within the data collection period, only four patients were recruited: two following discharge home and two after transfer to a community hospital ward (see Table 2). No patients who were invited to take part declined involvement. The main reasons for non-recruitment were that during the study period, few frail older people transitioned from the acute wards, due to capacity issues in community hospitals or community healthcare teams, or because they also needed social care packages, which were delayed. The small numbers meeting the criteria illuminated the challenges of facilitating care transitions for frail older people from the acute wards, despite being an integrated health system. Some of the frail older people who transitioned from acute wards were not considered by ward staff to have the mental capacity to make the decision to participate, as defined by the UK's Mental Capacity Act [43], mostly due to dementia. Other frail older people transferred to the community hospitals were not considered well enough to participate by ward staff; the transfer process sometimes led to patients feeling exhausted and disorientated. The interviews lasted about 30 minutes each. Patients were asked about their length of stay in the acute ward, their transfer or discharge planning and their involvement, and their transfer or discharge experience.

### Ethical issues

Ethical approval was obtained from a National Health Service Research Ethics Committee and the University Research Ethics Committee; the healthcare system's Research and Development Committee also gave approval. All interview and focus group participants were given written information sheets and time to decide whether to participate and they all gave written consent. All data collected were anonymised and kept securely on password protected computers. The case study site is reported anonymously as part of the ethics and governance for the project.

### Data analysis

The audio-recordings were professionally transcribed. The data were analysed using Ritchie and Spencer's five-stage framework approach [44]. One research team member initially led the analysis and then worked jointly with a second team member on the final analysis. The research team critically reviewed the analysis at different stages. The first stage was to gain familiarisation with the whole data set through reading the transcripts and noting key issues. The second stage was to produce a thematic framework through integrating the concepts from the literature review with the key issues noted during stage one. In the third stage, the thematic framework was applied systematically to the transcripts, coding the data according to the framework, adding additional codes where appropriate. The codes were grouped into categories and then overarching themes. The fourth stage was to create charts using Excel spreadsheets: one chart for each main theme, with a row for each data source (e.g. interview, focus group) and columns for categories. The coded data, identified with its source, was inputted into the charts. In stage five, the charts were critically reviewed by the research team for patterns and associations, which informed the final analysis.

### Scientific rigour

Lincoln and Guba's [45] criteria of credibility, transferability, dependability and confirmability guided scientific rigour throughout the research process. Triangulation of sources and methods enhanced the credibility, and the detailed description of the case provided will assist transferability of the findings to other settings. To promote dependability of the data, the research team worked together on the topic guide development and liaised closely throughout the data collection period, maintaining an audit trail of decisions. The rigour of the data analysis was enhanced through the research team working closely together. The steps to achieve credibility, transferability and dependability together promoted confirmability.

## Findings

The findings presented are based mainly on the key staff interviews and focus group data, as the patient sample size was so small. However, data from patients’ interviews are included where applicable and often concurred with staff perspectives. There are three themes presented: Care transitions within a vertically integrated healthcare system; Interprofessional communication and relationships; and Patient and family involvement in care transitions. Data sources are identified as: patient (P), key staff interview (K), and focus group (FG), with the number of the interview or focus group following (e.g. P1, K5, FG6).

## Care transitions within a vertically integrated healthcare system

Some staff participants expressed that a vertically integrated healthcare system facilitated care transitions for example:

The very fact that we're an integrated organisation, so we've got community hospitals as part of [the system], is in its nature, a really positive step forward so we haven't got this separation between the acute episode of the pathway of care and then the rehabilitation/onward method of care. (K7)

One initiative introduced since the integration was that a community liaison nurse provided an in-reach service at each acute hospital:

Working with teams to say ‘yes, actually we could take this lady home’, we could give her a couple of visits by the district nurse and we'll put that in place and that will get her through that next stage. (K4)

There had been substantial service reconfiguration with care pathways that aimed to provide a more integrated, patient-centred and responsive experience as patients moved between hospital and community (K14). For patients who had had a stroke, there was an early supported discharge team, which was considered to be responsive to patients’ needs (FG9). However some participants considered that further work was needed on the pathways:

I know integration will improve, there's been an awful lot of work, I am convinced about ACH [Adult Community Healthcare] teams aligning to that concept and that model, I just don't think we've got the model set up to work in the way that probably we need it. (K6)

Staff participants identified a lack of capacity within both the integrated healthcare system and social care services that affected care transitions:

I've been up to [acute ward] this morning and it's chocabloc with people waiting for packages of care, community hospitals, waiting, waiting – nursing homes, residential homes, and that's not uncommon. (K15)

Some patients who were waiting for a community hospital bed, therefore, remained in acute hospital wards to undergo rehabilitation and:

You often find by the time the bed comes up, is available, the patient's improved so much on the ward with what we've done with them they're actually able to go home.(FG2)

Conversely, the community hospital staff expressed that although their main remit was rehabilitation this was sometimes disregarded as ‘*There's just so much pressure to clear the beds in the acute’* (FG6). Thus, patients were sometimes transferred to community hospital wards when they were still medically unwell and some were then transferred back to an acute ward for further treatment, which increased their number of transitions (FG1, 3, 6, 7). Some patients experienced several transitions between different acute wards: ‘*The week before last I moved I think between three and four times in the week’* (P2). Her experience was not uncommon; focus group participants too discussed that frail older people were frequently moved between acute wards, while waiting to move to community hospitals or community healthcare at home. They expressed that these moves affected recovery (FG4), were ‘*disrespectful*’ (FG6), and that patients ‘*get more confused because of it and disorientated’* (FG7).

Some participants pointed out the limitations of the integrated system as it did not include mental health services and social care (K5, K14), which were often necessary for frail older people's continuing care and support (FG5). Participants discussed delays from the referral stage to organisation of social care, with patients on acute wards spending: *‘weeks waiting for a package of care from the social services’* (FG2) and there was a risk that their condition deteriorated while waiting (FG4).

## Interprofessional communication and relationships

Daily facilitated multidisciplinary meetings had been introduced to the acute hospital wards for reviewing patients’ progress and planning transitions. Participants commented that this development improved communication and relationships within the acute hospital wards, for example:

We're far more a team than we ever were before which is very helpful I think. If we need family meetings, before they would take up to a week or so to organise, whereas now we just say, ‘Shall we have a family meeting?’ (FG5)

Some acute hospital staff believed that communication across settings had improved since the integration and better relationships were developing:

I do think it's [communication] got better than it was since we integrated […] I've got to know people and I think more communication on the phone, like now I've met people from [names 2 community hospitals] so it's building up a trust between us. (FG8)

However, some community hospital staff expressed different views:

Sometimes we don't think we're given the correct information, because I think there is this fear that it might put the community hospital off from taking them [the patient], and this is where it falls down, because you lose that trust between the staff.(FG6)

Other staff expressed that there still needed to be better understanding about each other's roles across acute and community settings:

We have joined together as a [system] and I think there doesn't appear to be – they would probably say the same thing about us – our understanding of what the acute does and their understanding of what we [community] do, doesn't seem to be at the moment, as good as it could be. (K10)

As in the previous quote, community-based staff often referred to the acute wards as ‘*the acute’*, which implied separateness, rather than being an integrated system. They described frustrations about communicating with acute hospital staff, for example: difficulty contacting professionals, inadequate or illegible information on referral documents, and different computer patient record systems used in different settings and by different professionals. Assessment documents conducted in acute wards did not always accompany patients to community hospitals so families were sometimes asked for the same information again, which delayed the next stage (FG1, 6). In addition, it was expressed that acute hospital staff rarely communicated with community healthcare professionals about their patients:

I'm always surprised that some of our very complex patients, multiple morbidities, social problems, psychological problems, cognitive problems, go into a hospital and the hospital never thinks to get in touch and say, ‘what's this person like, what are their real problems?’ (K 2)

Community hospital staff and community healthcare teams also expressed that acute ward staff lacked understanding about their resources, and about patients’ home circumstances (FG3, K13) and others expressed that acute staff were not confident about community healthcare, for example:

If you're attracted to acute care and you like the ‘I'm doing obs and I'm doing rescue stuff’, you find it hard to believe it's right to send your patient to something calmer.(K5)

However, no such views were expressed by acute staff; the key issue from their perspectives was community hospital and healthcare capacity. One key staff member, who was formerly community-based, considered that community staff lacked understanding of how busy acute wards were: ‘*seeing how hard these staff work, how frustrated they are, how rushed, they don't have the time* [for planning care transitions]’ (K15). It was suggested that rotation of staff between acute and community provision could help staff understand services and roles in different areas (K5).

## Patient and family involvement in care transitions

Highlighting strategic commitment to involvement, a key staff member said that:

Patients and families should be involved in every discharge or transfer because that's our policy. (K1)

Staff participants repeatedly emphasised the importance of communicating with patients and families: ‘*put the patient first and speak to the family*’ (FG4). Examples of effective communication with patients and families included regular discussions and updates with patients and families, and early identification of a date for transition:

We've got to meet and greet and risk assess on our first contact with the patient and their family, so finding out if they're managing at home and then trying to make a rapid judgement of their place of discharge. (K 5)

We [occupational therapists, physios] speak to the relatives quite a lot on the phone and obviously see them when we see the patients, so we do have quite good relationships with the relatives, you know, if they want to be involved, and even for treatment and things. (FG4)

However, other participants expressed that, usually due to time pressures, communication with patients and families was sometimes lacking:

I think sometimes patients have good discharge planning, where they know exactly what's happening, however if there is a shortage of beds, I think it's up and out, that's my impression. (K 17)

One patient (P1) and her nephew described effective communication about her discharge and the discharge process, but the other three patient participants indicated a lack of involvement. One woman said:

One day the nurse came to me and she said ‘oh I've just been told I've got to take you to [community hospital]’. Well they only told me then, and then we had to go off within what, 10 minutes or so. (P2)

However, she believed that there was someone higher than the ward staff directing transitions: *‘the people who were giving the orders’* and:

The nurses didn't know any more than what I did I don't think. They weren't told [about the transfer], not the nurses. (P2)

One patient described conflicting information about whether he would be discharged home or transferred to a community hospital (P4). He felt that: ‘*my point of view wasn't even asked for’* and said: *I wasn't aware of anyone planning my discharge’*. Another patient said that she expected to be transferred to a community hospital as she observed other patients being transferred but: ‘*It would've been nice if they just come and explain it to you rather than just bump, bump, bump’*. (P3). Community hospital focus group participants discussed similar experiences:

I find that there's a lot of patients and their families who didn't know the patient was going to be referred to the ward.(FG1)

Several participants highlighted the complexities associated with working with families and the need for their early involvement. Families varied in how much they wanted to be involved however, which was often influenced by their previous relationship:

I think at the end of the day it depends how they are kind of related, was the network really between them. (FG4)

There was sometimes patient and/or family reluctance about discharge; underlying reasons included adjustment to changes in health and function, and family anxiety about continued, and sometimes increased, expectations to provide support:

The admission may have been the final straw for some carers and families who are struggling to cope for a long time before the admission. (K2)

If somebody comes in and is going to be discharged or transferred with quite a significant change in their pre-admission state, sometimes that's a mourning for family to have to work with, sometimes it's a massive adjustment in their own personal lives. (K5)

## Discussion

This study contributes by offering insights into care transitions from acute hospital wards specifically within a vertically integrated health system in England. The strengths of the research were the eliciting of views from staff in varied roles across the system, which illuminated barriers and facilitators for care transitions at the different levels identified in previous research [36]. While the patient sample gave some valuable perspectives of transitions at individual and interpersonal level [36], a limitation of the study is the very small sample. Baumann et al. reported problems in recruiting participants from a similar population [46]. The recruitment difficulties confirmed that many frail older people were remaining in acute hospital wards for lengthy periods and illuminated the complexity of these patients, as many were neither well enough, nor had the mental capacity, to be able to participate in an interview. The study took place in one area of England and explored experiences in one integrated healthcare system only; other integrated healthcare systems may differ in their service provision and facilities.

Ham et al. argued that, due to demographic changes, the division between primary care and secondary care is increasingly unhelpful [7]. Some participants in the current study could perceive benefits in being a vertically integrated organisation, without the traditional divide between hospital and community, which could resolve the reported communication issues between different healthcare providers at organisational level [9, 38]. Some staff expressed that integration with social care could further support transitions as delays were often social care-related. In England it has been announced that health and social care must become fully joined up and coordinated by 2018 [47] providing further health policy support to integration.

Howarth et al. suggested that for successful integrated care, there needs to be role awareness and effective communication between professional groups within teams [48]. However, in the current study, boundaries at staff level evidently remained; community staff often perceived the acute hospitals as being separate from their services and they believed that acute ward staff did not understand community provision and roles, nor appreciate patients’ home circumstances. The integrated system had been in place for only two years and the time taken to achieve effective integration is a core theme in the literature on vertical integration [49]. Coleman and Berenson point out that staff have often not worked within the settings to which they are sending patients, and so they may indeed be unfamiliar with their services [50]. The benefits of facilitating a regular dialogue between team members are well recognised [51–53] and can raise awareness of service availability [51] but in the case study site, there was no established multidisciplinary forum for acute hospital staff to meet with community hospital staff and community healthcare teams. Therefore, as in a US-based study [33], staff lacked formal opportunities to develop trusting relationships with colleagues in other settings and gain insight into their roles and care services. The newly instigated multidisciplinary daily facilitated meetings in the acute wards in the case study site, illustrated how a regular forum could promote effective communication and interprofessional relationships, which supported care transition planning. One of the case study site's initiatives since transition was for community professionals to provide liaison between community healthcare and acute hospital wards. This new role resembled the ‘In-reach Interface Model’ which is particularly suitable for older adults with ‘complex’ discharge needs [32], and could support better communication across settings. Other communication issues were the lack of shared information technology and electronic and written communication between settings, which were also highlighted as barriers in a national evaluation of integrated care projects in England [51].

The current study's results revealed delays in care transitions from acute wards but also frequent moves of older people between acute wards. Lack of capacity in community health and social care provision was perceived as an on-going barrier to transitions from acute wards; capacity or resource issues are acknowledged within the diverse reasons for delayed transfers of care in England [19, 27]. As in previous research [28, 31], participants expressed concerns that frail older people were at risk of deteriorating and acquiring infections during their long stays in acute wards. The current study also highlighted that frail older people were frequently moved between acute wards, an issue that has been raised for several years in England [17, 46, 54, 55] and the US [23]. The formation of a quality relationship between practitioners and frail, older people and their relatives supports positive transition experiences [10] but frequent moves affect relationship-building. The root cause of frequent moves may be that traditional acute hospital provision, which is focused on cure, does not meet the needs of frail older people who do not ‘fit’ this goal, but services should adapt to better meet the current and future population's needs [13, 54].

The importance of involving patients and supporting family carers has been previously highlighted [23, 26] and in the current study, a strategic commitment to patient and family involvement was affirmed. At ward level, staff participants discussed how they involved patients and families in planning for transitions but findings also revealed some lack of communication and involvement, a finding that supports previous studies [8, 11, 39, 56–58]. Decisions about transitions were often made quickly to increase bed availability in acute wards due to capacity issues across the system, but a quick discharge may affect the quality of service that staff can offer [28] and patients can be left feeling worried, dissatisfied and distrusting [20]. Nurses could be in a good position to support and empower patients as they transition between hospital and long-term settings [39] but the current study and previous research [9, 33] indicates that ward staff may lack control in relation to care transitions and disempowered staff may not feel well placed to empower patients.

## Conclusion

This study focused specifically on care transitions for frail older people from acute hospital wards to community hospitals or community healthcare teams, within a vertically integrated healthcare system in England. The study's findings highlighted that frail older people often remained on acute wards longer than was beneficial for them, often due to capacity issues in community hospital and healthcare team services. The findings also revealed that most staff working directly with patients were based either in acute hospitals, or in community hospitals and healthcare services, and there were few opportunities for them to build relationships and to develop understanding of service provision and roles in other parts of the system. Although an integrated healthcare system provides an organisational structure to support integrated care and specifically care transitions, the removal of organisational boundaries does not necessarily reduce boundaries between staff at interpersonal level and enable staff in different settings to work together effectively. Opportunities for staff to rotate between settings, and the establishment of forums for staff to build relationships and develop understanding of others’ roles and of other settings and their services, could assist staff to work in a more integrated way and would be a useful area for future research. The need to develop pathways for frail older people that prevent repeated moves around acute hospital wards, and the importance of effective communication with patients and their families about transitions, are areas that clearly need addressing and are of international concern.

## Figures and Tables

**Table 1.**
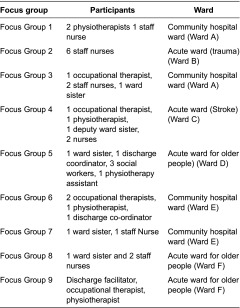
Focus groups and participants

**Table 2.**
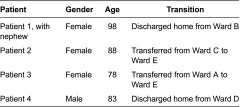
Patient interviews
